# Optimal treatment of breast cancer in women older than 75 years: a Korea Breast Cancer Registry analysis

**DOI:** 10.1007/s10549-019-05426-2

**Published:** 2019-09-06

**Authors:** Ye Won Jeon, Sun Hyong You, Jong Eun Lee, Hyun Jo Youn, Woosung Lim, Jai Hong Han, Chan Heun Park, Yong Seok Kim

**Affiliations:** 1 Department of Surgery, St. Vincent’s Hospital, College of Medicine, The Catholic University of Korea, Suwon, Korea; 2 Department of Surgery, Park Surgrcal Clinic, Suwon, Korea; 3 Department of Surgery, Soonchunhyang University Cheonan Hospital, Soonchunhyang University College of Medicine, Cheonan, Korea; 4 Department of Surgery, Chonbuk National University Medical School, Jeonju, Korea; 5 Department of Surgery, Ewha Womans University Medical Center, Seoul, Korea; 6 Department of Surgery, Center for Breast Cancer, Reaserch Institute and Hospital, National Cancer Center, Goyang-si, Korea; 7 Department of Surgery, Kangbuk Samsung Hospital, Sungkyunkwan University School of Medicine, Seoul, Korea; 8 Department of Surgery, Uijeongbu St. Mary’s Hospital, The Catholic University of Korea, #271, Cheonbo-ro, Uijeongbu-City, Gyenggi-Do 11765 Republic of Korea

**Keywords:** Breast neoplasms, Elderly women, Adjuvant treatment, Survival

## Abstract

**Purpose:**

The aim of this study was to investigate the therapeutic efficacy of adjuvant modalities for elderly Asian breast cancer patients using population-based data from the Korean Breast Cancer Registry database.

**Methods:**

We identified 53,582 patients who underwent curative surgery between January 2005 and December 2010. The primary end point was the comparison of overall survival between the administration or omission of adjuvant treatment modalities, including endocrine treatment, radiation therapy, and chemotherapy, in the elderly group (older than 75 years) compared with the control group (younger than 75 years).

**Results:**

Of the 53,582 patients analyzed, the total number of elderly patients was 901 (1.7%), and the number of control patients was 52,681 (98.3%). Although elderly patients were found to have larger tumor sizes (*p *= 0.024) and higher pathological stages (*p* < 0.001) than the control group, elderly patients were less likely to undergo adjuvant treatment compared to the control group. However, use of endocrine treatment in elderly patients with HR-positive breast cancer is associated with improved overall survival (OS) (adjusted OR 0.417; 95% confidence interval [CI] 0.240–0.726; *p *= 0.002). Furthermore, chemotherapy was associated with a significant improvement in OS in patients with stage II and III breast cancer (adjusted OR 0.657; 95% CI 0.462–0.934; *p *= 0.019).

**Conclusion:**

Endocrine treatment and chemotherapy for elderly patients are associated with improved OS. Therefore, personalized decision-making based on the potential survival benefit of adjuvant treatment modalities should be made with the careful counseling of all elderly patients with breast cancer.

## Introduction

Life expectancy has increased in recent decades due to developments in public health and medical science. Individuals aged 65 years and older are the fastest growing segment of Western regions, with people over the age of 75 years projected to comprise a disproportionate part of the USA population by 2030 [1, 2]. This phenomenon is also occurring in Asia. According to the Statistics Korea, life expectancy of a 75-year-old women was 11.6 years in 2005 and has extended up to 13.9 years in 2016 [3]. As the population life expectancy increases, the proportion of elderly patients with various malignancies has increased.

Breast cancer, which is the most commonly diagnosed cancer in Western and Korean women, is no exception to this trend. Incidence rates increase with age, with over 30% of cases diagnosed in women over 70 years in Western regions [1, 4]. According to reports from Statistics Korea(KOSTAT), the proportion of breast cancer diagnoses in women aged more than 70 years was 5.2% in 2004, and this proportion increased to 8.8% in 2015 [5]. However, despite the increasing proportion of elderly patients with breast cancer, standard therapeutic guidelines for this patient population are inconsistent, leading to challenges for clinicians in managing elderly patients. From previous publications, clinicians who manage elderly breast cancer patients should consider their functional status, comorbidities, clinical stages, biological characteristics of the cancer, and life expectancy [6–8]. The results of these considerations may lead to the under-treatment of elderly patients compared with younger patients [6, 9, 10]. Clinicians who manage elderly patients are required to choose the proper adjuvant modalities, such as endocrine treatment, radiation therapy, and chemotherapy, to maximize the efficacy of cure and maintain disease free status while, at the same time, minimizing interruptions in quality of life and unexpected early death.

Moreover, there are certain differences between Asian and Western regions with respect to age-specific incidence rates of breast cancer [11]. As the peak incidence age (35–64 years) of breast cancer in Korea is younger compared with Western regions (aged 65 year and older) [11, 12], the incidence of breast cancer in women older than 75 years at diagnosis is relatively low in Korea. Because the subgroup of patients older than 75 years would be too small, individual hospital-based registries would be inadequate to effectively address proper adjuvant modalities in elderly patients.

Therefore, the aim of this study was to investigate the characteristics of elderly breast cancer patients (aged 75 years and older) compared with non-elderly patients (control patients, less than 75 years old) and the therapeutic efficacy of adjuvant modalities (endocrine treatment, radiation therapy and chemotherapy) for elderly Asian breast cancer patients using population-based data from the Korean Breast Cancer Registry database.

## Methods

### Korean Breast Cancer Registry (KBCR)

The KBCR database was previously described in detail [13]. Briefly, the KBCR database is a Web-based, prospectively maintained nationwide database managed by the Korean Breast Cancer Society (KBCS). One hundred and two institutions have voluntarily participated in this registry since 1997. Before inserting personal information, along with various datasets, written informed consent is mandatory from the patient. From the initial conception of the KBCR database, principal investigators from every institution have agreed on the principles and processes of utilizing this database for research purposes. Essential registry items include patient age, sex, surgical method used, and breast cancer stage according to the seventh edition of the American Joint Committee on Cancer classification [14], biological status (hormone receptor [HR] and human epidermal growth factor receptor 2 [HER2]) and adjuvant treatment (endocrine treatment, radiation therapy and chemotherapy). The Korean Central Cancer Registry provides only mortality data, and the KBCR does not include information on tumor recurrence. According to the guidelines of utilizing the KBCR database, this study was approved by the Institutional Review Board (IRB) of Uijeongbu St. Mary’s Hospital, Catholic University of Korea (UC18RCSE0045). The KBCS approved our research objective and request for data in March 2018.

**Fig. 1:**
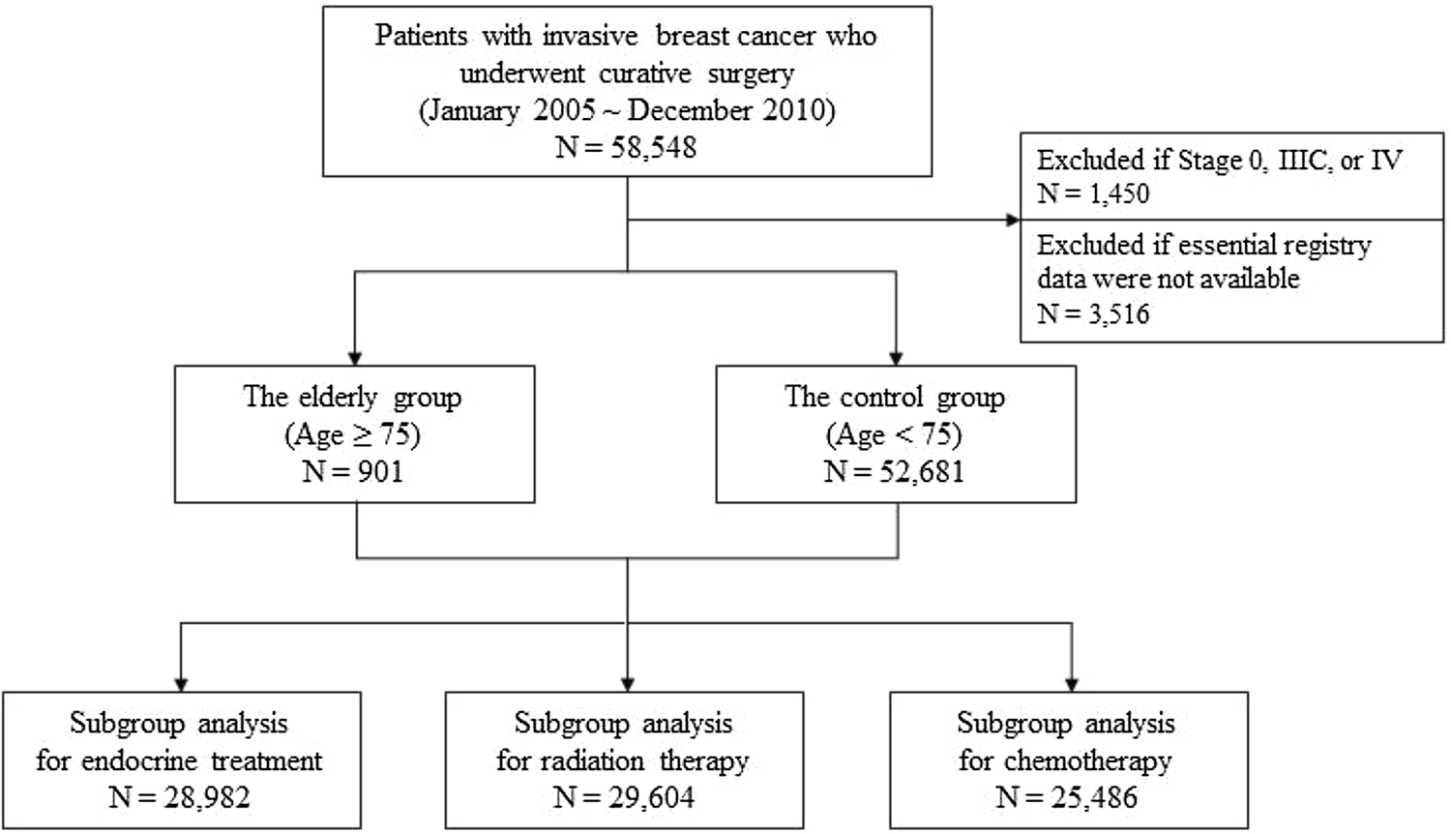
Schematic diagram of the study

From the KBCS registry, we assessed female patients with invasive breast cancer who underwent curative surgery between January 2005 and December 2010. To achieve a more accurate analysis, we excluded patients treated with neoadjuvant therapy and patients for whom essential registry data (gender, age, and cancer stage) were not available. Patients with distant metastasis (stage IV) and advanced stage (stage IIIc) at the time of diagnosis were excluded because these stages have the worst prognosis compared to other prognoses and serve as confounding factors for survival analysis. See Fig. 1 for a detailed list of excluded cases.

Data on the remaining 53,582 patients were included in the final analysis. All patients were categorized into one of two subgroups based on their age: elderly patients (≥ 75 years old) versus control patients (< 75 years old).

### Statistics

The means and standard deviations of physiological and demographic characteristics were calculated. A *t* test was used to compare the means when there were 2 categories. Proportions were compared using two-way tables and Chi-square tests. Survival curves were estimated using the Kaplan–Meier method, and log-rank tests were performed for comparison of survival curves. The primary end point was the comparison of overall survival between the administration and omission of adjuvant treatment modalities, including endocrine treatment, radiation therapy, and chemotherapy, in elderly patients. Overall survival (OS) was defined as the time from initial diagnosis of primary breast cancer to death from any cause. Multivariate analyses were conducted using a Cox proportional hazard regression model to study the effects of each therapeutic modality (endocrine treatment, radiation therapy and chemotherapy) on OS. Parameters included in the multivariate analysis model were as follows: breast surgery type, breast cancer stage, endocrine treatment, radiation therapy, and chemotherapy. All tests were two-sided, and a *p* value < 0.05 was considered to be statistically significant. All statistical analyses were performed using IBM SPSS software version 22.0 for Windows (IBM Corp., Armonk, USA).

## Results

### Patient characteristics

According to the eligibility criteria, we identified 53,582 patients who underwent curative surgery between January 2005 and December 2010 from the KBCS registry. The total number of elderly patients was 901 (1.7%), and the number of control patients was 52,681 (98.3%). Clinical and pathological characteristics are listed in Table 1.

**Table 1:** Clinical and pathologic characteristics of patients

Characteristics	Elderly group (≥ 75 years old)	Control group (< 75 years old)	*p* value
	N = 901	N = 52,681	
	No. (%)	No. (%)	
Age (years)			
Mean ± SD	78.39 ± 3.39	49.23 ± 9.80	
Median (range)	77.00 (75–94)	48.00 (20–74)	
75–78	561 (62.3)		
79–83	264 (29.3)		
84–	76 (8.4)		
Type of breast surgery			< 0.001
Breast-conserving surgery	300 (33.3)	29,304 (55.6)	
Mastectomy	586 (65.0)	22,507 (42.7)	
Excision only	8 (0.9)	267 (0.5)	
Unknown	7 (0.8)	603 (1.2)	
Type of axillary surgery			0.005
No operation	130 (14.4)	5777 (11.0)	
ALND	426 (47.3)	24,747 (47.0)	
SLNB	170 (18.9)	11,357 (21.6)	
SLNB → ALND	171 (19.0)	10,265 (19.4)	
Unknown	4 (0.4)	535 (1.0)	
Tumor size, cm			0.024
Mean ± SD	2.280 ± 1.74	2.017 ± 3.35	
Stage			< 0.001
I	381 (42.3)	27,715 (52.5)	
IIA	297 (33.0)	14,198 (27.0)	
IIB	120 (13.3)	5988 (11.4)	
IIIA	83 (9.2)	4424 (8.4)	
IIIB	20 (2.2)	356 (0.7)	
Intrinsic type			< 0.001
HR (+)/HER2(-)	431 (47.8)	23,799 (45.2)	
HR (+)/HER2(+)	41 (4.6)	4712 (8.9)	
HR (−)/HER2(-)	127 (14.1)	7008 (13.3)	
HR (−)/HER2(+)	69 (7.7)	5071 (9.6)	
Unknown	233 (25.8)	12,091 (23.0)	
Follow-up, months			< 0.001
Mean ± SD	69.79 ± 25.26	77.70 ± 22.44	
Median (range)	69 (5–134)	77 (5–119)	
Death event	317 (35.2)	4934 (9.4)	< 0.001

*SD* standard deviation; *ALND* axillary lymph node dissection; *SLNB* Sentinel lymph node biopsy; *HR* hormone receptor; *HER2* human epidermal growth factor receptor 2

The median age of patients in the elderly group was 77 years (range 75–94 years) and that of the control group was 48 years (range 20–74 years). Compared with the control group, the elderly group was more likely to undergo mastectomy (*p* < 0.001) and was less likely to undergo axillary surgery (*p* = 0.005). Elderly patients were found to have larger tumor sizes (2.280 ± 1.74 cm vs. 2.017 ± 3.35 cm, *p *= 0.024) and higher pathological stages (*p* < 0.001) than the control group. HR and HER-2 status were different between the elderly and control groups, with the elderly group having a higher rate of the HR(+)/HER2(−) subtype (*p* < 0.001).

The median follow-up for survival analysis was 69 months (range 5–134 months) in the elderly group and 77 months (range 5–119 months) in the control group. Three hundred and seventeen (35.2%) cases experienced death in the elderly group, and 4934 (9.4%) cases experienced death in the control group, during the study period. The cause of death in the elderly group is listed in Table 2. Despite the 157 cases of missing data for cause of death, the most prevalent cause of death in elderly patients was breast cancer (98 cases, 61.3% of all deaths), and the second most prevalent cause of death was other malignant disease (23 cases, 14.4% of all deaths).

**Table 2:** Causes of death in elderly patients in the Korean Breast Cancer Registry

Cause of death	Case (n)	Case (%)
Breast cancer	98	61.3
Respiratory failure	5	3.1
Cardiovascular disease	8	5.0
Brain hemorrhage/infarct	5	3.1
Kidney failure	5	3.1
Diabetes	4	2.5
Other malignant disease	23	14.4
Senility	4	2.5
Trauma	3	1.9
Suicide	1	0.6
Unknown	157	49.5
Total	317	

The life expectancy in elderly patients was analyzed and is shown in Table 3. Age ranges were subdivided into three categories (75–78, 79–83, and ≥ 84 years) according to patient distribution (60.7%, 29.6%, 9.7% each). Mean life expectancy of the entire elderly group was 121.51 months, which is approximately 10 years.

**Table 3:** Mean life expectancy and mean survival time of elderly patients

Age range	No. of patient (%)	Event	Mean life expectancy (months)	Mean survival time (months) (Kaplan–Meier survival analysis) (95% CI)	*p* value
75–78	547 (60.7)	170	137.90 ± 10.52	104.09 ± 2.03 (100.11–108.02)	< 0.001
79–83	267 (29.6)	97	104.86 ± 10.19	92.06 ± 2.36 (87.44–96.68)	
≥84	87 (9.7)	50	69.06 ± 13.32	68.76 ± 4.11 (60.69–76.82)	
Total	901 (100)	317	121.51 ± 24.98	100.03 ± 1.63 (96.85–103.22)	

*CI* confidence interval

### Role of endocrine treatment in elderly HR-positive breast cancer patients

To examine the therapeutic efficacy of adjuvant endocrine treatment in HR-positive patients, we extracted data for adjuvant endocrine treatment in HR-positive patients. From the registry, 471 patients in the elderly group and 28,511 patients in the control group were HR-positive breast cancer patients (Table 1). Among these patients, adjuvant endocrine treatment was administered to 342 (72.6%) patients in the elderly group and 26,292 (92.2%) patients in the control group. When compared with the control group, HR-positive elderly patients were less likely to undergo adjuvant endocrine treatment (72.6% vs 92.2%, *p* < 0.001).

**Fig. 2:**
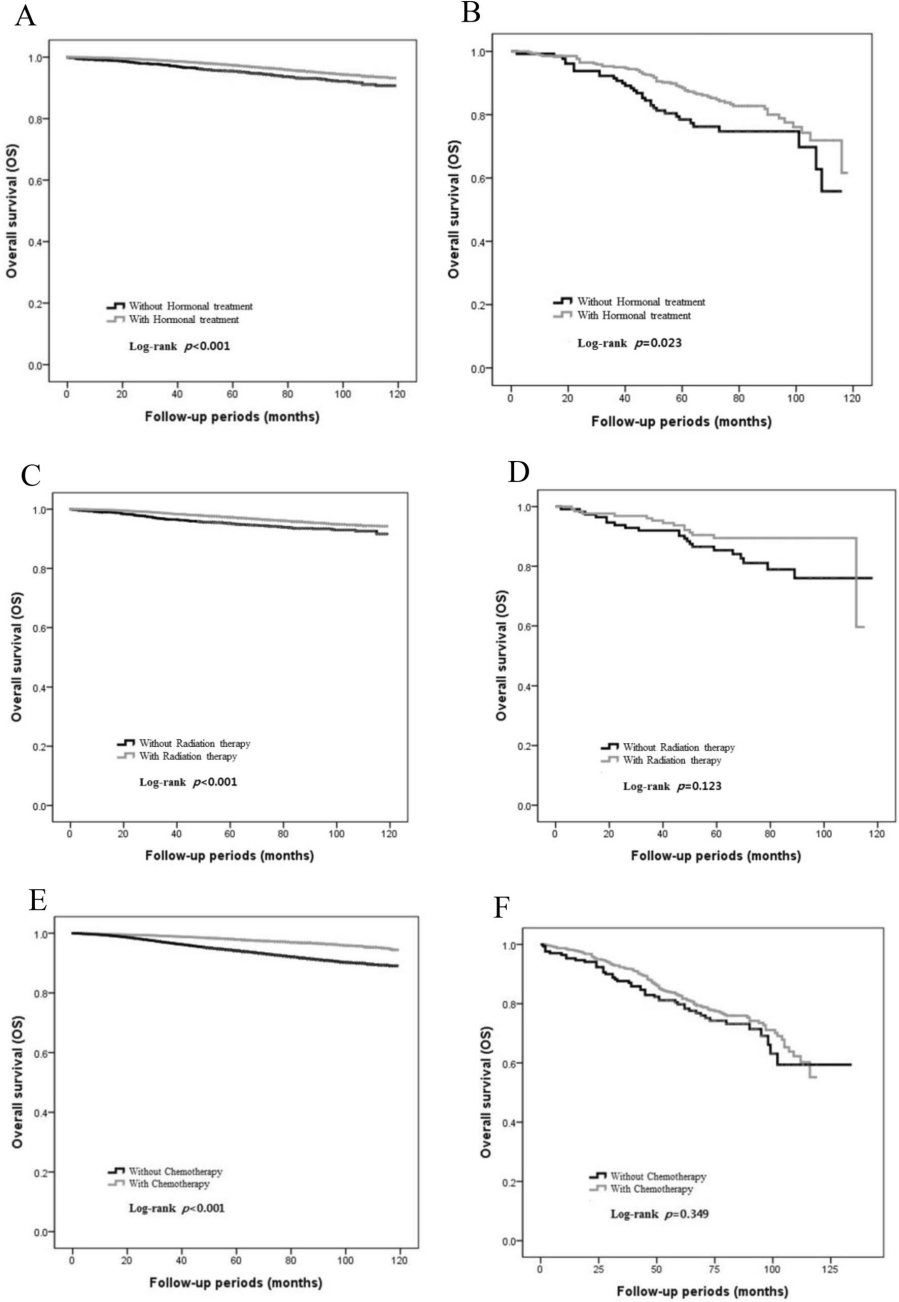
Overall survival of the control (< 75 years old) and the elderly (≥ 75 years old) groups by adjuvant modalities (hormonal therapy, radiation therapy and chemotherapy). **a**, **c**, **e**: control group; **b**, **d**, **f**: elderly group

Notably, in the survival analysis (Fig. 2a, b), adjuvant endocrine treatment conveys statistically significant overall survival gains in both groups (elderly group: *p *= 0.023; control group: *p *< 0.001). Furthermore, adjuvant endocrine treatment was an effective treatment option in elderly HR-positive breast cancer patients based on the multivariate analysis (adjusted OR 0.417; 95% CI 0.240–0.726; *p *= 0.002) (Table 4).

**Table 4:** Multivariate analysis by Cox proportional hazard model in the elderly group

Variable		OR	95% CI	*p* value
Type of breast surgery	BCS	1		
	Mastectomy	1.844	1.206–2.819	0.005
	Excision	6.299	2.526–15.705	<0.001
TNM stage	Stage I	1		
	Stage II	1.878	1.278–2.759	0.001
	Stage III	4.928	3.127–7.764	<0.001
Hormonal treatment	No	1		
	Yes	0.417	0.240–0.726	0.002
Radiation therapy	No	1		
	Yes	0.467	0.209–1.040	0.062
Chemotherapy	No	1		
	Yes	0.657	0.462–0.934	0.019

*OR* odds ratio, *CI* confidence interval, *BCS* breast-conserving surgery

### Role of radiation therapy in elderly patients who underwent breast-conserving surgery

We analyzed the therapeutic efficacy of adjuvant radiation therapy in patients who underwent breast-conserving surgery for initial treatment in each group. In the elderly group, 300 patients underwent breast-conserving surgery (Table 1). Among these patients, 159 (53.1%) patients received adjuvant radiation therapy, and 141 (46.9%) patients did not. In the control group, 29,304 patients underwent breast-conserving surgery. Among these patients, 27,194 (92.8%) received adjuvant radiation therapy, and 2110 (7.2%) did not. Elderly patients were less likely to undergo adjuvant radiation therapy after breast-conserving surgery compared with the control group (53.1% vs 92.8%, *p* < 0.001).

In the survival analysis, radiation therapy had a statistically significant overall survival benefit in the control group (*p *< 0.001) (Fig. 2c). However, adjuvant radiation therapy after breast-conserving surgery was not significant for overall survival benefit in elderly patients (*p *= 0.123) (Fig. 2d). Furthermore, in multivariate analysis, adjuvant radiation therapy did not show statistically significant effects on prognosis (adjusted OR 0.467; 95% CI 0.209–1.040; *p *= 0.062), even in early-stage breast cancer (Stage I–II; OR 0.594, *p *= 0.258) (Table 4).

### Role of chemotherapy in elderly patients with advanced breast cancer

We analyzed the therapeutic efficacy of adjuvant systemic chemotherapy in patients with stage II and III disease in each group. Stage II and III breast cancer were identified in 520 patients from the elderly group and in 24,966 patients from the control group (Table 1). Among these patients, adjuvant systemic chemotherapy was administered to 129 (24.9%) patients in the elderly group and 14,655 (58.7%) patients in the control group. Elderly patients with stage II and III breast cancer were less likely to undergo adjuvant systemic chemotherapy compared with those in the control group (*p* < 0.001). This result was also consistent when classified by breast cancer stage (from IIA to IIIB) (Table 5).

**Table 5:** Mean survival analysis of study groups with or without chemotherapy according to breast cancer stage (AJCC 7th edition)

	Stage	Chemotherapy	Case (%)	Mean survival (95% CI) (Months, Kaplan–Meier survival analysis)	*p* value
Elderly group	Stage IIA	No	219 (73.9)	98.973 ± 2.559 (93.958–103.988)	0.482
		Yes	78 (26.1)	93.007 ± 4.563 (84.063–101.950)	
	Stage IIB	No	97 (78.3)	87.756 ± 4.275 (79.377–96.136)	0.733
		Yes	26 (21.7)	76.847 ± 6.857 (63.407–90.287)	
	Stage IIIA	No	39 (46.8)	71.312 ± 7.559 (56.497–86.127)	0.049
		Yes	44 (53.2)	89.457 ± 6.928 (75.879–103.035)	
	Stage IIIB	No	14 (68.8)	68.652 ± 10.471 (48.128–89.175)	0.059
		Yes	6 (31.2)	37.000 ± 3.362 (30.411–43.589)	
Control group	Stage IIA	No	1533 (10.8)	112.619 ± 0.631 (111.382–113.856)	0.185
		Yes	12,665 (89.2)	113.527 ± 0.199 (113.136–113.918)	
	Stage IIB	No	251 (4.2)	100.200 ± 2.495 (95.310–105.089)	< 0.001
		Yes	5737 (95.8)	110.295 ± 0.362 (109.587–111.004)	
	Stage IIIA	No	124 (2.8)	93.349 ± 3.845 (85.813–100.885)	< 0.001
		Yes	4300 (97.2)	105.019 ± 0.517 (104.006–106.031)	
	Stage IIIB	No	25 (7.1)	83.899 ± 7.686 (68.834–98.965)	0.091
		Yes	331 (92.9)	84.548 ± 2.611 (79.432–89.665)	

*CI* confidence interval

In the survival analysis, adjuvant systemic chemotherapy had a statistically significant overall survival benefit in the control group (*p *< 0.001) (Fig. 2e) but not in the elderly patients (*p *= 0.349) (Fig. 2f). Although the use of this treatment slightly improved overall survival in the elderly group with stage IIIA disease, the influence on survival of adjuvant systemic chemotherapy in the elderly group was not significant compared with results from the control group (Table 5). However, in the multivariate analysis, after adjusting for multiple confounders (breast surgery type, breast cancer stage, endocrine treatment, and radiation therapy), adjuvant systemic chemotherapy was an effective treatment modality that extended overall survival for elderly patients with stage II and III breast cancer (adjusted OR 0.657; 95% CI 0.462–0.934; *p *= 0.019).

## Discussion

Our study is of particular importance in light of the aging Asian population, especially in Korea, and a lack of randomized data to guide clinical decision-making for the treatment of elderly breast cancer patients. In this study, despite larger tumor sizes and higher pathologic stages occurring more often in elderly patients compared to non-elderly patients, adjuvant treatment (endocrine treatment, radiation therapy and chemotherapy) was omitted without impacting elderly patient survival. However, adjuvant endocrine treatment and chemotherapy were obviously associated with improved overall mortality for elderly breast cancer patients in population-based data from the Korean Breast Cancer Registry.

To provide more age-specific and personalized therapeutic options for elderly breast cancer patients, it is important to classify elderly patients with respect to clinical data. ‘How old is old?’ is a very difficult question to answer to distinguish an elderly person. ‘Old age’ is subjective terminology that is affected by many environmental and biological factors, such as the degree of the national healthcare system, socioeconomic status, and personal biological health, which varies by country. Furthermore, there are several difficulties in defining a numeric reference point for old age when analyzing clinical data. Life expectancy in one country can be a useful tool to represent elderly status in a society and is easy to establish as a numeric reference point when analyzing clinical data. Instead of ‘How old is the patient?’, ‘How long is the patient is expected to live?’ can suggest more objective and effective options when analyzing geriatric studies [15]. Therefore, having 10 years of natural life expectancy was considered old age (aged 75 years and older) in our study design.

Although the number of elderly breast cancer patients has increased over the last couple decades, major clinical trials and studies for breast cancer treatment are mainly focused on younger patients [15]. Such conventional study design causes elderly people to remain underrepresented in many clinical trials, resulting in difficulty in creating a therapeutic plan for elderly breast cancer patients. Elderly patients greater than 75 years of age are often regarded as a unique group of patients who are not willing to obey the standard therapeutic recommendations. Furthermore, under-treatment for both surgical and adjuvant treatment is more common in elderly patients than in younger patients. There are several reasons for under-treatment in elderly patients. First, old age is associated with an increased risk of systemic therapy-related toxicity. Second, tumor biology in breast cancers of elderly patients is somewhat different than in breast cancers of younger patient. Elderly patients tend to have more favorable breast cancer characteristics at diagnosis, including anatomical stage and phenotypic characteristics [7, 16, 17].

Endocrine treatments using aromatase inhibitor (AI) or tamoxifen are the most commonly used adjuvant systemic treatments in elderly patients with HR-positive breast cancer. This method has fewer complications and better compliance than any other treatment modality and is highly effective in reducing breast cancer recurrence and increasing overall survival in elderly HR-positive breast cancer patients. Although clinical studies of endocrine agents are limited in elderly patients, the median age of women enrolled in clinical trials using AIs as adjuvant treatment agents was often greater than 60 years because of the eligibility requirement of postmenopausal status [18–20]. Furthermore, these studies definitively demonstrated superior long-term efficacy and safety of AI as an initial adjuvant treatment for postmenopausal women with HR-positive breast cancer. In addition, the results from the Early Breast Cancer Clinical Trialists’ Collaborative Group (EBCTCG) overview demonstrated decreased risk of breast cancer recurrence and death in women aged 70 years and older with early-stage HR-positive breast cancer receiving 5 years of tamoxifen, similar to that observed in younger patients [21]. This result was consistent with our current study, which found a survival benefit of adjuvant endocrine treatment for elderly patients with HR-positive breast cancer.

Radiation therapy is a highly effective method to destroy cancer cells and to reduce local recurrence in the breast or nearby lymph nodes that may persist after breast surgery. Although adjuvant radiation therapy after breast-conserving surgery is considered standard of care, radiation therapy does not improve overall survival for many elderly breast cancer patients, as reported in previous studies [22, 23]. Furthermore, a meta-analyses of radiotherapy trials by the EBCTCG showed that the 5-year absolute reduction rate in local recurrence associated with radiation therapy was 17% in younger and 18% in older age groups [24]. Therefore, the National Comprehensive Cancer Network guidelines suggest that adjuvant radiation therapy after breast-conserving surgery may be omitted for breast cancer patients who are older, have limited life expectancy, or have favorable tumor characteristics [15]. Although the current study did not demonstrate a risk reduction benefit for adjuvant radiation therapy on local recurrence in elderly patients, our study reveals that adjuvant radiation therapy after breast-conserving surgery is not statistically significant for overall survival benefit in elderly patients by multivariate analysis.

Similar to young patients, chemotherapy is an important systemic adjuvant treatment option for elderly breast cancer patients. However, research on the impact of chemotherapy on survival outcomes in elderly patients is limited, as clinical trials typically exclude this group. Furthermore, chemotherapy-related complications, including cardiotoxicity, acute myelogenous leukemia (AML)/myelodysplastic syndromes (MDS), and death, cause elderly patients to be under-treated [15, 25, 26]. Despite these limitations, meta-analyses of randomized trials by the EBCTCG demonstrated clear evidence of a benefit for patients receiving chemotherapy aged 50–69 years at diagnosis, especially those with estrogen receptor-poor tumors [27]. A recent analysis of retrospective English cancer registration data showed that chemotherapy is associated with improved breast cancer-specific survival in elderly women (aged 70–79 years) with early breast cancer at high risk of recurrence [28]. In our study, adjuvant systemic chemotherapy was an effective treatment modality that extended overall survival for elderly patients with stage II and III breast cancer.

Although we adjusted for all possible and available factors in our analysis, our study was limited by the information available in the KBCR database. First, this study carries a risk of selection bias, and the results were lacking information on locoregional recurrence, distant metastasis, and breast cancer-specific survival. Although we did not investigate the disease free survival rate according to adjuvant modalities (endocrine treatment, radiation therapy and chemotherapy) in elderly patients, we believe that analyzing these factors would not change the power of our study because the overall survival rate is an acceptable and powerful endpoint in oncology. Further limitations include the lack of specific data on endocrine treatments (regimens, treatment period, etc.), radiation therapy (dose, field, etc.), and chemotherapy (regimens, dosage, etc.). In addition, the reasons for under-treatment of elderly patients, including physician discretion, presence of underlying comorbid conditions, socioeconomic and lifestyle factors, and patient preference, could not be evaluated in this study due to limitations of the KBCR database. Finally, the ethnic homogeneity of the KBCR database may limit the generalizability of our findings to other racial and ethnic groups.

## Conclusion

In conclusion, using population-based data from the Korean Breast Cancer Registry database, this study shows that elderly patients (aged 75 years and older) with breast cancer are more likely than non-elderly patients (< 75 years old) to be diagnosed at a later stage of disease and are less likely to undergo adjuvant treatment, including endocrine treatment, radiation therapy and chemotherapy. However, use of endocrine treatment in elderly patients with HR-positive breast cancer and chemotherapy in elderly patients with stage II and III breast cancer are associated with improved overall survival. Although it is not possible to determine the survival benefit of adjuvant treatment without additional information on underlying comorbidities and functional status, these data suggest that endocrine treatment in elderly patients with HR-positive breast cancer and chemotherapy for elderly patients with stage II and III breast cancer are associated with improved OS. Therefore, physicians must undertake personalized decision-making for individual patients according to reasonable estimates of predicted life expectancy, effect of certain treatments on mortality, and side effects associated with particular treatments when counseling elderly patients with breast cancer.
